# Demystifying the New Dilemma of Brain Rot in the Digital Era: A Review

**DOI:** 10.3390/brainsci15030283

**Published:** 2025-03-07

**Authors:** Ahmed Mohamed Fahmy Yousef, Alsaeed Alshamy, Ahmed Tlili, Ahmed Hosny Saleh Metwally

**Affiliations:** 1 Instructional and Learning Technology, College of Education, Sultan Qaboos University, Muscat 123, Oman; 2 Educational Technology Department, Faculty of Specific Education, Fayoum University, Fayoum 63514, Egypt; 3 Educational Foundations and Administration, College of Education, Sultan Qaboos University, Muscat 123, Oman; a.alshamy@squ.edu.om; 4 Faculty of Education, Alexandria University, Alexandria 21500, Egypt; 5 Smart Learning Institute, Beijing Normal University, Beijing 100875, China; ahmed.tlili23@yahoo.com; 6 Educational Technology Department, Faculty of Education, Helwan University, Cairo 11795, Egypt; ahmed_hosny@edu.helwan.edu.eg

**Keywords:** brain rot, Gen Z, Gen Alpha, doomscrolling, Oxford Word of the Year, digital addiction, working memory, cognitive load

## Abstract

**Background/Objectives**: The widespread phenomenon of “brain rot”, named the Oxford Word of the Year 2024, refers to the cognitive decline and mental exhaustion experienced by individuals, particularly adolescents and young adults, due to excessive exposure to low-quality online materials, especially on social media. The present study is exploratory and interpretative in nature, aiming to investigate the phenomenon of “brain rot”, with a focus on its key pillars, psychological factors, digital behaviors, and the cognitive impact resulting from the overconsumption of low-quality digital content. **Methods**: This study employs a rapid review approach, examining research published between 2023 and 2024 across PubMed, Google Scholar, PsycINFO, Scopus, and Web of Science. It explores the causes and effects of brain rot, focusing on the overuse of social media, video games, and other digital platforms. **Results**: The findings reveal that brain rot leads to emotional desensitization, cognitive overload, and a negative self-concept. It is associated with negative behaviors, such as doomscrolling, zombie scrolling, and social media addiction, all linked to psychological distress, anxiety, and depression. These factors impair executive functioning skills, including memory, planning, and decision-making. The pervasive nature of digital media, driven by dopamine-driven feedback loops, exacerbates these effects. **Conclusions**: The study concludes by offering strategies to prevent brain rot, such as controlling screen time, curating digital content, and engaging in non-digital activities. Given the increasing prevalence of digital engagement, it is essential to explore a variety of strategies, including mindful technology use, to support cognitive health and emotional well-being. The results can guide various stakeholders—policymakers, practitioners, researchers, educators, and parents or caregivers—in addressing the pervasive impact of brain rot and promoting a balanced approach to technology use that fosters cognitive resilience among adolescents and young adults.

## 1. Introduction

On 2 December 2024, “brain rot” was chosen as Oxford’s Word of the Year. This term refers to “the supposed deterioration of a person’s mental or intellectual state, especially viewed as the result of overconsumption of material (now particularly online content) considered to be trivial or unchallenging. Also: something characterized as likely to lead to such deterioration” [1]. The symptoms of brain rot include spending hours on-screen, anxiety when away from mobile devices, and a weakened ability to pay appropriate attention to valuable activities [2]. Satici et al. [3] suggested that while brain rot is not a formally recognized medical condition, it is very real, particularly among younger generations, i.e., the Gen Z Post-Millennial Generation (born 1995–2009) and Gen Alpha (born after 2010), who are doomscrolling and navigating an ever-more-connected, screen-centered world. Over 4 billion internet-connected young adults spend 6.5 h a day online, many of whom passively watch low-value content on social media and the internet [4].

Entertainment used to involve participation; today, instant gratification provided by digital platforms like TikTok encourages users to seek more and more stimulating content to achieve the same level of satisfaction. The algorithm, for example, behind the “For You Page” on TikTok encourages an endless loop of consumption that can promote desensitization and shortened attention spans [5]. The overstimulation of such pages/digital platforms ultimately causes one to struggle with the ability to consume longer, uncut content and further depletes meaningful involvement in real-life experiences [6]. Such consequences of digital addiction have even been more visible across growing generations that have enjoyed technology as their source of entertainment. Excessive screen time also gives rise to mental health issues, such as social withdrawal, distorted perceptions of reality, and increased levels of anxiety and depression [7]. It has also been linked to potential risks for cognitive health, particularly in adolescents and young adults, which include impaired brain development; increased risk of cognitive, behavioral, and emotional disorders; and potential early onset of dementia in late adulthood [8]. Overall, excessive screen time is associated with negative outcomes, such as lowered self-esteem, increased incidence and severity of mental health issues and addictions, slowed learning and acquisition, impaired concentration, memory issues, and an increased risk of premature cognitive decline [8,9].

Although a link has been established between digital addiction and cognitive deficits, with impairments of memory and attention being commonplace, much of the literature does not link such symptoms to the specific digital behaviors that are possibly exaggerating the brain rot phenomenon [10]. More fundamentally, there is a limited understanding of the particular digital behavior of brain rot, its effects on cognitive functions, and how brain rot varies across generations, specifically in Generation Z and Generation Alpha, who have been raised in a predominantly digital world. While the psychological and cognitive effects of screen time have been researched, as in [2,4], the broader social and emotional impacts like social withdrawal, distorted perceptions of reality, and diminished capacity to engage in real-life experiences remain less well known. Moreover, effective strategies to counteract brain rot are yet to be explored, especially those that would appeal to adolescents, younger adults, and relevant stakeholders.

No research study, to the best of our knowledge, has systematically investigated the concept of brain rot and what might lead to it. To address this research gap, the present study aims to systematically explore the phenomenon of “brain rot”, focusing on psychological factors, digital behaviors, and cognitive impacts resulting from the overconsumption of low-quality digital content. It investigates the underlying causes of brain rot, its effects on cognitive functions, and the strategies that can help mitigate its detrimental impacts. Specifically, this study addresses the following research questions (RQs):

RQ 1. What factors contribute to the recognition of “brain rot” as a phenomenon among adolescents and young adults?
RQ 2. What specific digital behaviors are closely associated with the onset of brain rot?
RQ 3. How does brain rot affect cognitive functions such as memory, attention span, and problem-solving abilities in adolescents and young adults?
RQ 4. What strategies do adolescents and young adults find most effective in mitigating the effects of brain rot?

## 2. Background

Henry David Thoreau, in his book *Walden*, published in 1854, launched the term “brain rot” among communities for the first time. Thoreau’s reference, though unrelated to technology, captures the essence of the condition of a gradual atrophy in the capacity to think critically, concentrate, and be in the world appropriately [11,12]. Because it refers to a state of intellectual deterioration that is independent of age, education, and culture, the phrase “brain rot” is not a novel idea. In other instances, it may even be connected to the intellectual stagnation that some groups of people go through. The digital age, in which millions of movies are widely shared, particularly among young people, closely aligns with this concept [13]. Short-form videos, designed for entertainment and rapid dissemination across social media platforms, frequently include content related to body shaming, promotion of unhealthy eating habits, and unsubstantiated and potentially dangerous health remedies. Numerous researchers are investigating how the internet affects adolescents’ cognitive development through the widespread intake of shallow and occasionally hazardous content [14].

The majority of adolescents spend several hours every day glued to screens, often multitasking: scrolling through social media, checking instant messages, watching short videos, or gaming. This is one of the major sources of stimulation that drives brain rot [15]. Recently, considerable evidence from large-scale, extensive epidemiological studies and randomized controlled trials has offered a thorough picture of the ways in which internet use impacts social, cognitive, and psychological outcomes. The findings demonstrate that excessive exposure to irrelevant materials on the internet causes cognitive malfunctions in the brain [16].

A study by Li et al. [17] examined the impact of excessive exposure to continuous streams of information and emotions in gaming, particularly in relation to its effects on specific brain functions. The findings indicate that such overuse affects attentional abilities, as the constant influx of online information compels individuals to split their attention among multiple sources. Additionally, it influences memory processes and sleep quality and results in cognitive failures. These results highlight the importance of addressing procrastination and impulse control in order to enhance the quality of cognitive functions [18].

Researchers have attempted to evaluate addiction to dopamine in the brain. This neurotransmitter, as its name implies, is responsible for conveying feelings of pleasure or satisfaction associated with specific actions [19], including receiving a notification on social media or scrolling through a social media feed. As engagement on social platforms increases, so does the brain’s need for a dopamine hit. This creates a loop of perpetual engagement as the brain becomes addicted to the fleeting gratification that comes from new information, likes, or comments [20].

## 3. Research Method

**Figure 1:**
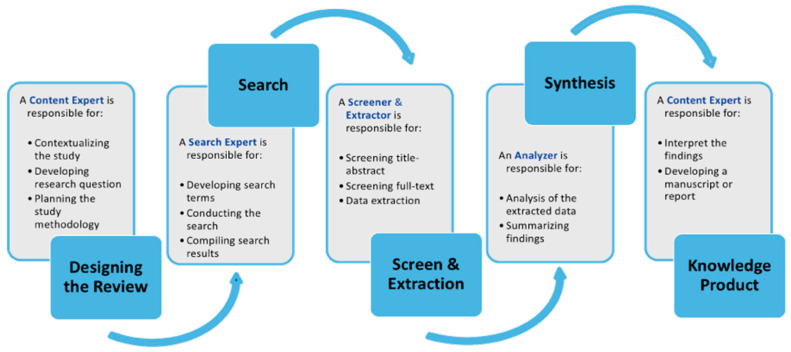
Steps of conducting a rapid review [23].

To provide a thorough understanding of “brain rot” and its association with digital behavior, cognitive functions, and mitigation strategies, we conducted a rapid review [21]. This is “a form of knowledge synthesis in which components of the systematic review process are simplified or omitted to produce information in a timely manner” [22] (p. 1). A rapid review is employed when immediate evidence is required to inform decisions, particularly in rapidly changing domains such as public health and digital behavior. Unlike conventional systematic reviews, rapid reviews streamline some of the steps involved, such as data extraction and quality appraisal, in order to present insights faster, without compromising relevance. Rapid reviews are particularly useful when resources or time are constrained, with a view to offering a brief summary of current evidence while maintaining transparency and minimizing bias [22,23]. Using this approach, a search was conducted within the period of 2023 to 2024 according to Google trends in interest over time when the term has flourished, which was specifically focused on uncontrolled and excessive use of digital platforms by adolescents and their effects on the cognitive health of the adolescent brain. Figure 1 depicts the steps for conducting this rapid review [23].

The first search, through the selected databases—PubMed, Google Scholar, PsycINFO, Scopus, and Web of Science—identified 381 records. After removing duplicates (112 records) and the exclusion of another 15 records due to their not being written in English, 254 records needed to be screened for relevance.

**Figure 2:**
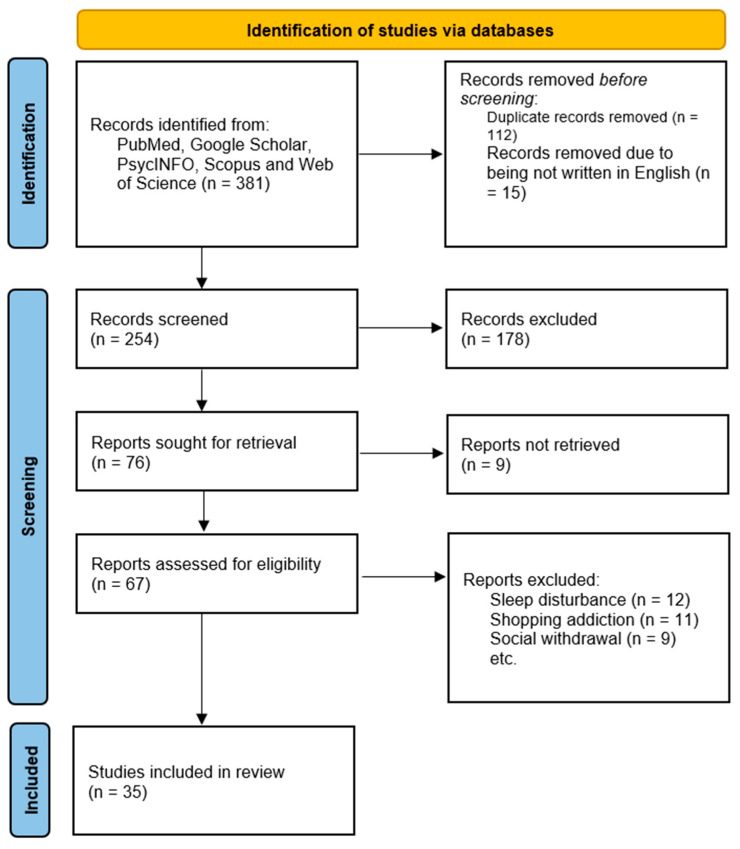
Data selection process.

Out of these 254, 178 records were excluded after screening, which resulted in 76 papers being sought for retrieval. Of these, 9 papers were irretrievable, which then left 67 papers that were assessed for eligibility. Inclusion criteria were used to select studies that were directly related to concepts such as “brain rot” and cognitive decline due to overuse of digital technology, particularly among adolescents and young adults. Exclusion criteria were set to eliminate studies that were unrelated to this topic, such as articles on sleep disturbances, shopping addiction, or social withdrawal. This multi-step process was effective in ensuring that the final studies were relevant and methodologically sound, adding considerably to the insights into the cognitive impacts of excessive screen time. Finally, 35 papers were included in the review. Figure 2 illustrates the data selection process according to the PRISMA flow diagram.

Table 1, below, presents the number of studies related to each search term per publication year. It can be seen that the term “brain rot” did not appear explicitly/verbatim in any database, likely due to its novelty from an academic perspective. Moreover, the term “cognitive decline” was mentioned in 29% of the articles, with 21% in 2023 and 8% in 2024. This reveals that the topic remained relevant throughout the period and that it was more prominent in the earlier year. Additionally, Table 1 shows that the most-discussed topic was “excessive screen time”, which appeared in 57% of the articles, with 34% in 2023 and 23% in 2024. This indicates its continued relevance but a slight decrease in focus over time. The term “digital addiction” appeared in 71% of the articles, with 43% in 2023 and 29% in 2024, showing that despite a small decline in 2024, the subject has been consistently emphasized. The term “doomscrolling” was less frequently addressed, appearing in 17% of the articles, mainly in 2023 (11%), which underlines its niche status compared to broader topics like screen time and addiction. Finally, “zombie scrolling” was the least discussed; it appeared in 11% of the articles, 9% in 2023 and 2% in 2024, highlighting its minor role in the research landscape concerning digital behaviors.

### 3.1. Data Analysis

#### 3.1.1. Text Analysis

For the analysis and synthesis of data from the selected literature, Voyant Tools (https://voyant-tools.org/, accessed on 20 January 2025), a web-based reading and analysis environment for digital texts, was employed [24]. The software enabled the following:

- Text Mining Analysis: This study delved into the common themes, terms, and behaviors linked to “brain rot” and cognitive decline. The analysis focused on the frequency and context of the following words: “attention span”, “cognitive decline”, “digital addiction”, “doomscrolling”, and “zombie scrolling”.
- Contextual Clustering: Clustering of related terms and concepts, identifying commonalities across studies, particularly those which linked specific digital behaviors to cognitive outcomes, was performed.
- Trend Analysis: The way “brain rot” and other related cognitive effects have been discussed and might have shifted within the literature published between 2023 and 2024 was assessed.

#### 3.1.2. Thematic Synthesis

After conducting the text analysis, the results were organized into thematic categories according to the aforementioned research questions as follows:

1. Factors of Recognition of “Brain Rot”: The identification of demographic, social, and psychological factors that influence how adolescents and young adults perceive and recognize the phenomenon of “brain rot”.
2. Digital Behaviors Associated with “Brain Rot”: The levels of individual behaviors related to doomscrolling and zombie scrolling in relation to cognitive disengagement or “brain rot” were ascertained.
3. Cognitive Functions Affected by “Brain Rot”: It was established qualitatively and quantitatively exactly how brain rot specifically affects the memory, attention span, and problem-solving abilities of adolescents and young adults according to the 35 studies included in the review.
4. Mitigation Strategies: The studies related to the strategies used by adolescents and young adults to mitigate the effects of brain rot include digital detoxing, mindfulness, and cognitive training.

## 4. Results and Discussion

This study was exploratory and interpretative in nature. Thus, this section is structured according to each of the research questions. It presents and discusses the findings from the four research questions about the phenomenon of “brain rot” in adolescents and young adults, namely, the factors that contribute to its recognition, the digital behaviors linked to its onset, its effects on cognitive functions, and the strategies that adolescents and young adults employ to counteract the condition.

### 4.1. Factors Contributing to the Recognition of “Brain Rot” Among Young Adults

**Figure 3:**
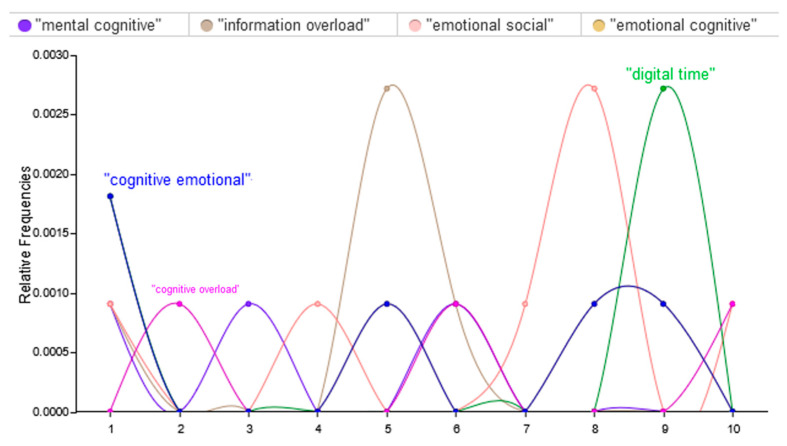
Relationship between the most frequently repeated words for factors that contribute to the recognition of “brain rot”.

The recognition of brain rot as a phenomenon is shaped by the amount and nature of digital engagement [25]. In this respect, overwhelming consumption, especially by adolescents and young adults, of social media, video streaming, and instant messaging may lead to mental fatigue. By analyzing the relationship between the most frequently repeated words, it was found that several terms were found to affect the clarity of brain rot, as depicted in Figure 3, namely, mental cognitive, information overload, emotional social, and emotional cognitive. Figure 3 reveals the relative frequencies of the four keywords: “mental cognitive”, “information overload”, “emotional social”, and “emotional cognitive” within a predefined range, depicting notable trends in their usage as Term Frequencies (TFs). Each TF represents the raw frequency of a specific term in the corpus, while the Document Count (DC) on the x-axis indicates the number of terms across all documents. A Relative Frequency is calculated by dividing the TF by the DC, typically expressed as a percentage. Each colored curve illustrates ups and downs in the popularity of these terms; noticeable peaks denote periods of higher relevance. For instance, the “information overload” curve is spiky, which means that information overload will continue to worsen in this social media era, wherein adolescents and young adults are bombarded with an overwhelming volume of content daily. This usually contains low-quality information that may mask meaningful insights and make credible sources hard to discern. This might heighten anxiety and decrease attention span, which could reduce the ability to process and engage with the wealth of information available. In contrast, the “emotional social” and “emotional cognitive” curves display more consistent yet moderate patterns of usage, signifying ongoing engagement with these themes, but without the same intensity or rapid shifts.

Based on the thematic analysis, three main factors were identified that contribute to brain rot, namely, excess screen time, addiction to social networking, and cognitive overload. Each is detailed below.

#### 4.1.1. Excess Screen Time

The amount of time young adults spend in front of screens on a daily basis is one of the primary causes of the growing reputation for brain rot. Deyo et al. [26] demonstrated that college students spent an average of 7 h per day using mobile devices for entertainment purposes, not counting time spent on them for academic purposes. The results showed that screen time was associated with increased levels of anxiety, depression, and stress among students [26]. Therefore, it may be beneficial to promote strategies that encourage students to engage in activities that are not related to screen time. Cognitive overload may arise as a result of such multitasking; mental exhaustion and a lack of attention are caused by the brain’s difficulties in digesting and remembering information [27,28].

Recent research indicates that younger adults are hooked on such activities, such that they increasingly report brain rot-like symptoms, such as poor concentration, a sense of mental cloudiness, and diminished cognitive function [29]. The brain is so overstimulated with switching between tasks that it finds difficulty focusing on important or meaningful activities. As a result, both productivity and motivation go down [30].

#### 4.1.2. Addiction to Social Networking

The widespread influence of social media has been elevated to the forefront of the list of critical elements taken into account when diagnosing brain rot in young adults. Platforms like Facebook, Instagram, and TikTok are designed to keep users’ attention and encourage constant scrolling, which can result in hours of use with no real breaks [5]. The dopamine-driven feedback loop reinforces this habit; young people get a rush of satisfaction when they scroll through their feeds, which makes them want to scroll even more [28]. This compulsive behavior may eventually turn into an addiction-like pattern when young adults reach the flow state, where the demand for digital engagement becomes excessive and it becomes increasingly difficult to stop using these platforms. Brain rot is further exacerbated by the emotional toll of social media [31].

There is a tendency to experience constant feelings of discontentment, depression, and anxiety due to overstimulation with information, the pressure of impressionable lifestyles, and beauty standards that constantly surround us, and this results in a narrative of “social media brain rot”. This insight is crucial because young adults are capable of recognizing these issues and categorizing them effectively [27].

#### 4.1.3. Cognitive Overload

The overwhelming amount of information that we constantly receive in our everyday lives is another reason that young people are experiencing brain rot [32]. The internet is a very real source of cognitive overload in the form of news, entertainment, and social media updates [16]. Social media data and online course updates are additional sources that the brain is desperately trying to process, leading to mental fatigue and a decline in certain cognitive functions. Previous research has indicated that prolonged periods of digital engagement, especially among young adults, are associated with greater difficulty in focusing and sustaining attention [33].

Cognitive load can be categorized into three types: intrinsic, extraneous, and germane [34]. Intrinsic load is the difficulty of processing information, which varies according to the complexity of the material. The load is constant and is unaffected by external conditions. Conversely, extraneous load is a matter of how information is presented and the relative ease or difficulty of processing it. Finally, there is germane load, the mental effort involved in integrating new information into pre-existing knowledge frameworks, called schemas. This refers to the mental effort required to process new information and store it in permanent memory and is vital in learning and understanding at a deep level [35]. Beyond brain rot, overwhelming exposure to digital content, especially through social media and multitasking, can lead to extraneous load, as the presentation of information tends to be extremely dispersed and overwhelming [15]. This constant cognitive load, along with diminished focus and mental clarity, can further impair the brain’s ability to manage both intrinsic and germane loads effectively, resulting in mental fatigue and cognitive decline. When overwhelmed, the ability to process information meaningfully is hindered, which can lead to signs of cognitive decline among young adults.

### 4.2. Digital Behaviors Associated with Brain Rot

**Figure 4:**
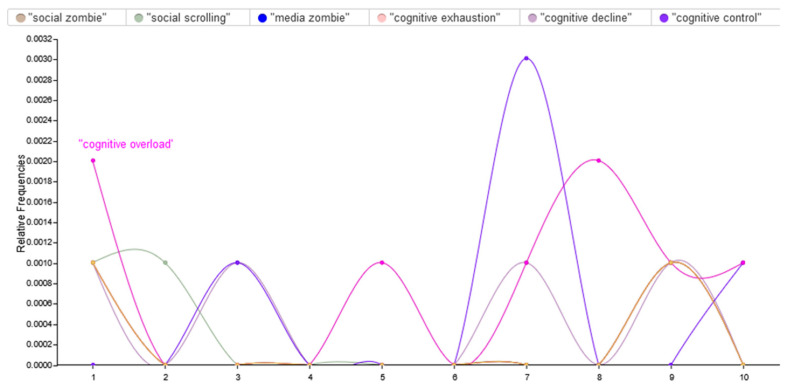
The relationships between digital behaviors and cognitive control and decline.

Brain rot is increasingly associated with a variety of specific digital behaviors, many of which have become common among young adults in the digital age [36]. These behaviors, including doomscrolling, zombie scrolling, and social media addiction, are highly related to all negative cognitive and emotional impacts of excessive screen time [37]. It is very important to understand how these digital behaviors lead to the development of brain rot for prevention and intervention purposes [38]. To explore these connections, we conducted a text analysis to identify the relationships between digital behaviors and cognitive control and decline. In Figure 4, the different heights and trends of curves indicate the patterns of use associated with the terms, that is, certain trends in “brain rot” digital behaviors. For instance, the curves for “cognitive exhaustion” and “cognitive decline” are steep, with the concepts at times highly salient, no doubt reflecting increasing concerns about popular fears of decreased mental acuteness due to excessive screen time and low-value content. On the other hand, the terms “social scrolling” and “media zombie” have more incidental mentions, indicating their relevance but in a manner that is less consistently emphasized in the discourse. In general, this analysis shows the complex interaction of these digital behaviors and points to how overindulgence in low-quality media contributes to cognitive fatigue and reduced acuteness of the mind.

#### 4.2.1. Doomscrolling

The compulsive action of scrolling through low-quality content from social media feeds or news websites that focuses on negative or distressing information has been termed doomscrolling [3]. While it may present a small, temporal sensation of being current, its detrimental effects on cognitive health are rather large [33]. This behavior is particularly concerning because it leads to heightened levels of anxiety, stress, and secondary traumatic stress, which have been linked to reduced mental well-being [39]. The constant consumption of negative news not only exacerbates emotional distress but also contributes to a mental state of rumination, where individuals repeatedly focus on distressing content, preventing them from engaging in more productive or calming cognitive activities [40].

Cognitively, doomscrolling places individuals in a state of hyper-vigilance, where they are constantly drawn to threatening content. Research by Starvaggi et al. revealed that approximately 52% of the information in the most viewed TikTok videos consists of misinformation [41]. Constant stimulation and mental engagement are similar to a form of cognitive overload, which can be particularly dangerous in young adults whose cognitive control is still developing [15]. This behavior hampers the brain’s capacity to process and integrate information efficiently, resulting in a gradual decline in cognitive abilities due to fatigue over time.

#### 4.2.2. Zombie Scrolling

Zombie scrolling is the term used to describe how people passively scroll through social media or websites without any purpose other than, perhaps, occasional meaningful interaction. A lack of intentionality or motive is the crucial component of zombie scrolling, possibly induced in a kind of dissociative state of mind [42]. The usual evidence for this behavior might be a general loss of control over the activity, which is propelled by the search for novelty from a brain that is pleased with a constant spurt of digital stimulation. According to Jiang et al. [43], despite the commonness of zombie scrolling and the fact that it does not involve the emotional distress that characterizes doomscrolling, it results in some cognitive depletion nonetheless. The mindless consumption of digital content can reduce the brain’s capacity for sustained attention and focus, which are key elements of cognitive performance [44]. This behavior not only leads to a decrease in cognitive engagement but also fosters a sense of detachment from reality, exacerbating feelings of loneliness and emotional disconnection [45].

#### 4.2.3. Social Media Engagement Loops

Another significant concern is social media addiction. Various social media platforms are addictive, like Instagram, TikTok, and Facebook, each notifying the user continuously of distracting topics that divert attention and contribute to cognitive fatigue [46]. Their visually stimulating interfaces combined with stimulating sound alerts encourage prolonged screen time, often leading users to spend excessive hours engaged with these platforms. The addictive nature of these platforms, sometimes referred to as “slot-machine feedback loops”, can lead to compulsive checking, whereby individuals feel an uncontrollable urge to stay connected and informed, even at the expense of their mental health [47].

The constant use of social media leads to a continued need for attention and cognitive resources. This perpetual engagement interferes with the brain’s ability to focus on more meaningful or important tasks, leading to diminished productivity and cognitive decline [48]. Additionally, neurochemical gratifications associated with social media, such as those produced by dopamine, reinforce this behavior, and therefore it is hard for users to disconnect, despite awareness of the damage being caused to their mental health. Over time, this behavioral addiction enhances the propensity toward brain rot by means of chronic overstimulation and cognitive fatigue manifested as reduced mental clarity, focus, and emotional stability.

#### 4.2.4. Emotional and Cognitive Control in Digital Behavior

Long-term exposure to the rapid, fragmented nature of digital content interferes with the brain’s capacity for efficient emotional management and information processing [15]. This effect is particularly important in adolescents, for whom skills of cognitive and emotional regulation are still in the development stage [44]. Excessive use of digital devices, especially prolonged immersion in games, leads to increased impulsivity, a reduction in the ability to concentrate, and problems with decision-making, all of which are factors that contribute to brain rot [49].

Moreover, excessive digital exposure shrinks the ability to invest in more restorative intellectual activities, such as deep thinking, reflection, or focused problem-solving [50]. This, cumulatively, will result in a severe decline in cognitive function and will make everyday tasks that require sustained attention and mental effort much more difficult to navigate.

### 4.3. Impact of Brain Rot on Cognitive Functions in Adolescents and Young Adults

**Figure 5:**
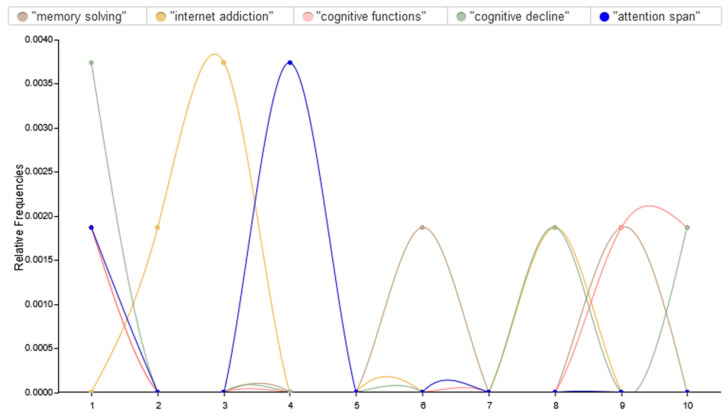
Relationship between internet addiction behaviors and cognitive poverty among young people.

Text analysis reveals that internet addiction behaviors are closely associated with cognitive decline, particularly in areas such as memory, attention span, and problem-solving abilities, highlighting the impact of excessive digital use on cognitive function, as depicted in Figure 5. These curves show the different emphases on various cognitive aspects related to brain rot. For example, the “cognitive decline” and “attention span” curves are high, indicating high levels of concern about these issues at one point in time, probably due to continuous talk about how excessive digital engagement affects the cognitive abilities of young adults. On the other hand, the “internet addiction” curve is stable, reflecting the consistent awareness of its relevance to cognitive health. The “memory solving” curve is less pronounced, which could mean that, though it is an important aspect, it is not discussed as much in the discourse.

The thematic analysis further reveals four main areas of brain rot in relation to cognitive function, namely, distorted memory, attention span, problem-solving abilities, and cultural and social considerations. Each of these is detailed below.

#### 4.3.1. Distorted Memory

Studies have shown that too much exposure to digital media disrupts the consolidation and retrieval processes of memory. For example, Mendez et al. [15] showed that internet addiction disrupts cognitive control, which is supposed to facilitate memory. The constant distraction caused by notifications and the speed at which one consumes information prohibits deep processing that may allow for long-term retention. Again, this finds resonance with the work of Jiang et al. [43], who have highlighted that internet addiction is associated with deficits in working memory, which is considered important for learning and retaining information.

#### 4.3.2. Attention Span

Brain rot destroys yet another cognitive function, namely, attention span, as the pervasive use of smartphones and social media establishes an environment of rapid intake of information, leading to only superficial engagement with content. This phenomenon, more popularly known as “doomscrolling”, has been said to fragment attention and reduce one’s ability to focus for continuous periods of time [39]. Aarts et al. [51] discuss how these repeated interruptions might cumulatively lead to cognitive overload, thereby undermining the potential for sustained attention and leaving a greater potential for distraction.

#### 4.3.3. Problem-Solving Abilities

Both memory- and attention-related problem-solving abilities are also compromised in the case of brain rot among young adults [52]. Growing dependence on digital tools for information and decision-making decreases cognitive flexibility toward adapting to change and taking on new challenges. Impulsivity and poor decision-making abilities, common characteristics of problematic internet users, are likely to play an important role in problem-solving, as suggested by Emadi Chashmi et al. [44]. This, in turn, could lead to a preference for instant gratification associated with internet use, making individuals choose quick fixes over more in-depth, analytical thinking [53].

#### 4.3.4. Cultural and Social Considerations

The cultural context of adolescents’ and young adults’ use of technology further exacerbates these cognitive deficits. O’Reilly and Mohan [45] indicate that parental influence, along with societal norms, may allow excessive utilization of the internet and contribute to increased cognitive decline. Additionally, the interaction of internet literacy and addiction, as pointed out by Wei et al. [48], calls for educational interventions in the course of developing healthy digital habits in a way that could reduce cognitive impairments.

### 4.4. Effective Strategies for Mitigating the Effects of Brain Rot

**Figure 6:**
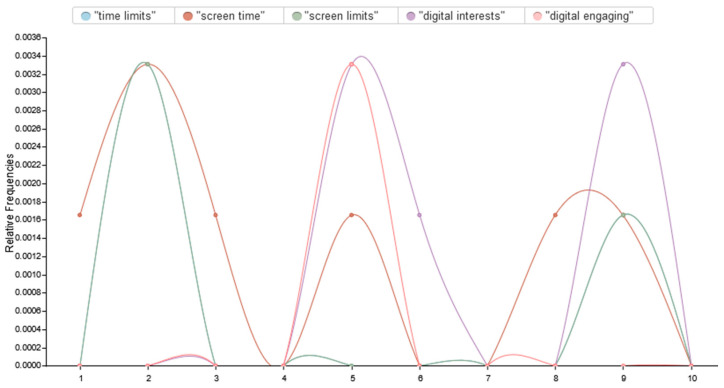
The correlational analysis of screen time and improved mental health and cognitive functions.

Our analysis recommended a number of key strategies recently suggested in the literature, along with implications for mitigating the impacts of excessive digital engagement. These include regulating screen time, curating media feeds, incorporating non-digital materials, and fostering social support and community engagement. The text analysis aligned with our categorized strategies, as the correlational analysis of screen time demonstrated improvements in cognitive abilities among young adults, as shown in Figure 6.

#### 4.4.1. Regulating Screen Time

The existing literature has documented those individuals who self-regulate their digital usage exhibit significant improvements in their mental health [15]. By tracking the amount of time spent on various types of digital platforms, individuals are often shocked by their own usage; this often serves as a wake-up call [54]. Establishing boundaries, such as reducing daily screen time and deleting distracting apps, can help restore cognitive balance. Jiang et al. [43] emphasize the importance of these boundaries in reducing cognitive overload, thereby enhancing focus and productivity.

#### 4.4.2. Curating Media Feeds

In a world where sensationalism abounds, adolescents and young adults learn to be very selective with their sources of information. This helps protect their mental space, as unfollowing accounts that provoke negative emotions and instead seeking uplifting content becomes a form of self-care [47]. This corroborates the finding of Taskin et al. [39] that a positive digital environment can reduce anxiety and depression, which are often exacerbated by negative news cycles.

#### 4.4.3. Incorporating Non-Digital Materials

Engagement in non-digital activities acts as an effective counterbalance to cognitive drain caused by excessive screen time [55]. These activities—music, writing, outdoor adventures, and volunteer work—not only provide a much-needed break from screens but also serve as stress relievers for the mind and emotional well-being. According to Emadi Chashmi et al. [44], having a variety of interests outside of screens could also increase cognitive flexibility and improve problem-solving. By investing time in hobbies or other unplugged activities they enjoy, young adults not only relax but also promote computational thinking skills [56], which are most effective in combating “brain rot”.

#### 4.4.4. Fostering Social Support and Community Engagement

In the processes of building social connections and developing supportive communities, the phenomenon of brain rot can be combated. According to O’Reilly and Mohan [45], positive social networking—both online and physical—helps mitigate feelings of isolation and loneliness, which are often associated with prolonged screen use. Building volunteer opportunities and participating in group activities not only provide a sense of belonging but also make an individual cognitively and emotionally more resilient. This also emphasizes the crucial influence of teachers and parents in regulating digital media usage.

## 5. Conclusions and Implications

Brain rot is, indeed, a growing concern among adolescents and young adults living in today’s high-tech world. Characterized by brain fog and decreased concentration, brain rot appears to be exacerbated by excessive screen time or overexposure to frivolous online content, ultimately leading to diminishing cognitive function. This research opted for a rapid review approach, synthesizing recent research published within the period 2023–2024 that looked into the uncontrolled and excessive use of digital platforms by adolescents and young adults and their effects on cognitive health. The behaviors to which brain rot is attributed, like doomscrolling, zombie scrolling, and social media addiction, have deep ramifications in terms of one’s mental health, emotional well-being, and conception of self. The criticality of their development phases make adolescents and young adults increasingly vulnerable to the crippling effects of digital overstimulation. This is especially brought about by the compulsive nature of technology, particularly the way dopamine-driven feedback loops associated with social media use amplify cognitive overload and emotional exhaustion. Indeed, research underlines the harmful or detrimental consequences these behavioral patterns have on executive functioning skills, such as memory, planning, and decision-making. Mindful approaches to the use of technology in mitigating brain rot involve limiting screen time, curating digital content, and engaging in non-digital activities, among other strategies, which remarkably improve cognitive health and emotional resilience. Moving forward, as society continues to evolve in its relationship with technology, it will be increasingly important to prioritize balanced media consumption and internet literacy.

The scope of this study is limited to the concept of “brain rot”. While there are many positive aspects of using digital media that can enhance various skills and improve overall communication, our manuscript focuses solely on the implications of low-quality content and its negative effects, particularly among adolescents and young adults.

Future research should be directed at effective interventions that would help adolescents and young adults form healthier habits with digital technologies. If awareness is raised and the necessary tools are provided to navigate the digital world, this vulnerable population can protect their cognitive functions and lead fuller lives. In such a way, policy makers, practitioners, researchers, educators, and parents or caregivers can direct their efforts to combat the omnipresent effects of brain rot and to work toward a more balanced, participatory approach to technology use in the modern era, which could enhance cognitive resilience.

## Figures and Tables

**Table 1:** The number of studies related to each search term per publication year.

Year	Brain Rot	Cognitive Decline	Excessive Screen Time	Digital Addiction	Doomscrolling	Zombie Scrolling	Total Articles
2023	0	7	17	21	6	4	23
2024	0	3	10	12	3	2	12
Total	0	10	27	33	9	6	35
